# Bioprospecting Indigenous *Oenococcus oeni* Strains from Chinese Wine Regions: Multivariate Screening for Stress Tolerance and Aromatic Competence

**DOI:** 10.3390/foods14071207

**Published:** 2025-03-29

**Authors:** Yongzhang Zhu, Xiaoqing Hong, Zhenghua Xu, Shuwen Liu, Kan Shi

**Affiliations:** 1 College of Enology, Shaanxi Engineering Research Center for Viti-Viniculture, Viti-Viniculture Engineering Technology Center of State Forestry and Grassland Administration, Heyang Experimental and Demonstrational Stations for Grape, Ningxia Helan Mountain’s East Foothill Wine Experiment and Demonstration Station, Northwest A&F University, Yangling, Xianyang 712100, China; zhuyongzhang@nwafu.edu.cn (Y.Z.); hongxq@nwafu.edu.cn (X.H.); 2 Guangdong Provincial Key Laboratory of Intelligent Port Security Inspection, Huangpu Customs District P.R. China, Guangzhou 510700, China; xuzhciq@163.com

**Keywords:** MLF, lactic acid bacteria, biosafety, stress tolerance, aromas

## Abstract

Malolactic fermentation (MLF), an essential enological process for wine deacidification and aroma development, is predominantly mediated by *Oenococcus oeni* (*O. oeni*). This investigation characterized 170 indigenous *O. oeni* isolates from two principal Chinese viticultural regions (Yinchuan, Ningxia, and Changli, Hebei) through polyphasic analysis. Forty-nine strains demonstrating genetic potential for efficient malate metabolism and biosafety compliance (absence of ethyl carbamate and biogenic amines genes) were subjected to adaptive laboratory evolution under enologically relevant stress conditions. Comparative evaluation with the superior indigenous strain SD-2a revealed eight stress-adapted isolates exhibiting superior MLF kinetics, completing L-malic acid degradation in Marselan wine. Solid-phase microextraction-gas chromatography-mass spectrometry (SPME-GC-MS) profiling identified three isolates’ (3-31, 9-10, and 9-50) significant enhancement of key fermentation aromas in experimental fermentations. These oenologically adapted indigenous strains demonstrate promising potential as regional-specific starter cultures, providing a scientific foundation for developing terroir-expressive winemaking practices and optimizing microbial resources in China’s wine industry.

## 1. Introduction

Malolactic fermentation (MLF) is a critical process in winemaking, contributing to the reduction of acidity, enhancement of flavor, increase in complexity, and improvement of microbial stability [1,2]. This process is primarily driven by lactic acid bacteria (LAB), with *Oenococcus oeni* (*O. oeni*) being the dominant species, particularly due to its tolerance to harsh wine conditions such as high alcohol content, low pH, and elevated SO_2_ levels [3,4,5]. The evaluation of stress resistance is a key criterion in selecting superior MLF strains [6,7]. Indigenous strain selection is particularly important, as adaptation to regional terroir significantly influences fermentation outcomes [4]. Notably, an alcohol content of 10% (*v*/*v*) and pH (about 3.5) are identified as critical stress factors affecting the survival of *O. oeni* [5,8]. The Ningxia and Changli regions represent prominent wine-producing areas in China. However, the market is currently dominated by imported *O. oeni* preparations, resulting in limited strain diversity and a subsequent homogenization of wine styles [9]. To mitigate these challenges and ensure successful MLF, the screening and identification of indigenous *O. oeni* strains with superior traits are imperative. In recent years, winemaking countries such as Spain, Italy, Argentina, and Chile have increasingly focused on developing and utilizing native *O. oeni* strains. These isolates demonstrate superior malolactic fermentation proficiency, pronounced resilience to environmental stresses, and advantageous biosafety characteristics, thereby maintaining the unique regional typicity of the wine [10,11,12,13,14,15,16].

The enzyme β-D-glucosidase derived from wine LAB plays a pivotal role in aroma modification, as it hydrolyzes glycosidically bound flavor precursors, releasing volatile aroma compounds and thereby improving wine quality. Importantly, significant variability in enzymatic activity has been observed among strains [17,18,19,20]. Extensive studies have focused on screening commercial strains with enhanced enzymatic activity based on the transcriptional expression levels of the β-D-glucosidase gene [21,22,23,24]. Esters represent another critical category of aroma compounds, formed and modified during different stages of MLF. Despite their relatively low concentrations, esters significantly impact wine aroma, particularly in relation to fruity descriptors and flavor complexity [25,26]. Additionally, insights into glutathione’s anti-stress mechanisms in *Saccharomyces cerevisiae* have been further elucidated through genetic and transcriptional analyses [27].

The presence of diverse LAB strains can also influence the metabolic risk profile of wines. Specifically, the production of ethyl esters and biogenic amines during MLF renders it a more challenging fermentation process to control compared to alcoholic fermentation (AF) [8,28,29,30]. During MLF, carbamates produced by arginine metabolism and biogenic amines, such as histamine, putrescine, and tyramine obtained from amino acid metabolism, affect the safety of fermented foods [31,32,33,34]. During MLF, the metabolism of arginine via the arginine deiminase pathway (ADI pathway) leads to the production of carbamates, including ethyl carbamate, which is formed through the reaction of urea with ethanol [35]. The key enzymes in this pathway, arginine deiminase, ornithine transcarbamylase, and carbamoyl kinase, are encoded by the *arcA*, *arcB*, and *arcC* genes, respectively, which collectively form the *arcABC* gene cluster [36,37,38]. Strains harboring this gene cluster are capable of arginine metabolism, while those lacking it are not [37,38,39,40]. Furthermore, biogenic amines such as histamine, putrescine, and tyramine, primarily derived from amino acid precursors including ornithine, histidine, and tyrosine, are synthesized through the action of decarboxylase enzymes encoded by the *hdc*, *odc*, and *tdc* genes, respectively [31,41,42,43]. It has been observed that different strains exhibit specificity in the synthesis of these biogenic amines [43,44]. The presence or absence of functional genes responsible for the synthesis of these risk substances serves as a critical criterion for screening superior commercial strains [10,21,45,46].

Emerging as a prominent player in the global wine industry, China boasts a multitude of wine-producing regions and a rich repository of microbial resources [47]. However, there remains a notable absence of superior local commercial strains. Hence, it is crucial to identify robust strains of *O. oeni* that can withstand high alcohol content and low pH levels, which is critical for enhancing the distinctiveness of Chinese wine production. The primary objective of this study was to identify the most suitable indigenous *O. oeni* strains through a multi-step approach: screening based on functional genes while excluding those with risk genes, selecting superior strains with stress resistance, and evaluating their oenological properties, including malic acid metabolism and aroma analysis.

## 2. Materials and Methods

### 2.1. Strains Isolation and Medium

Strains were isolated using spontaneous MLF from Cabernet Sauvignon and Cabernet Gernischt wines sourced from the Changli and Yinchuan regions of China. Using PCR species-specific amplification, a total of 222 isolates from the Changli region and 207 isolates from the Yinchuan region were identified as *O. oeni*. Among these, 170 representative strains were randomly selected for AFLP fingerprinting analysis at a 90% similarity level, comprising 82 strains from Changli and 88 strains from Yinchuan [48]. *O. oeni* SD-2a and 31-DH, preserved in the microbiology laboratory of the College of Enology at Northwest A&F University, were used as type strains [49,50,51,52]. These 172 strains were utilized for subsequent experimental analysis and discussion. During the various phases of MLF, wine samples (0.1 mL) were taken from a 10-fold dilution series in physiological solution and plated onto ATB medium (pH 4.8, 26 °C, glucose 10 g/L (Damao chemical Reagent Partnership Enterprise, Tianjing, China), yeast extract 5 g/L (Aobox Bio-technology Co.Ltd, Beijing, China), peptone 10 g/L (Aobox Bio-technology Co.Ltd, Beijing, China), MgSO_4_⋅7H_2_O 0.2 g/L (Xilong Science Co., Ltd, Shenzhen, China), MnSO_4_⋅4H_2_O 0.05 g/L (Xilong Science Co., Ltd, Shenzhen, China), cysteine/HCl 0.5 g/L (Biotopped Technology, Co., Ltd Beijing, China), and tomato juice 250 mL/L) supplemented with 28 g/L agar (Aobox Bio-technology Co.Ltd, Beijing, China) [53]. Single colonies were selected based on phenotypic differences among *O. oeni* strains and cultured in ATB medium under optimal conditions at 26 °C in a constant temperature anaerobic incubator for 3–8 days.

### 2.2. Targeted Screening by PCR of Functional Genes

Functional genes, including *bgl*, *estA*, and *gshR*, were screened to detect ethyl carbamate, while *arcA*, *arcB*, *arcC*, *hdc*, *odc*, and *tdc* genes were selected for detecting biogenic amines. PCR amplification of all genes was performed in a total volume of 25 μL by the primers in Table S1. The reaction system was as follows: 2.5 μL of 10 × PCR Buffer, 0.5 μL of dNTP mix, 1 μL of each primer (Forward and Reverse) with a 10 pmol/L concentration, 1 U of DNA Taqase with a 2.5 U/μL concentration, 14.4 μL of ultrapure water, and 4 μL of Genomic DNA. PCR program of genes was carried out with the following cycling parameters: initial 3 min denaturation at 94 °C, followed by 32 cycles of 30 s at 94 °C, 30 s annealing at 55 °C, 1 min extension at 72 °C, and final extension step of 10 min at 72 °C. The products were detected by 1.5% agarose gel electrophoresis and photographed under UV light using the Champ Gel 500 plus (Saizhi Technology Co., Ltd., Beijing, China).

### 2.3. Stress Resistance Analysis of O. oeni Strains During MLF

To establish standard growth curves, the OD_600_ values of SD-2a in simulated wine were monitored every 12 h until the stable phase was reached (data provided in Table S2). For stress tolerance evaluation, strains were inoculated into stress-simulated wine at a 4% (*v*/*v*) inoculation rate under anaerobic culture conditions at 20 °C. The experimental conditions included varying pH levels (3.8, 3.5, and 3.2) and ethanol concentrations (10%, 12%, 14%) (detailed in Table 1). By referencing the growth pattern of SD-2a, the relative growth of each isolated strain was determined through the calculation of ΔOD_600_, defined as the difference between the final OD_600_ reading at 72 h and the initial OD_600_ at 0 h.

### 2.4. MLF of O. oeni in Lab-Scale Vinification

Fermentation experiments were conducted using red grapes of the Marselan variety at laboratory scale in glass fermentation tanks with a 5 L working volume. AF was initiated by inoculating a commercial strain of *Saccharomyces cerevisiae* (CECA, Angel Yeast, China) into the must, which was maintained between 18 °C and 25 °C until the residual reducing sugar concentration decreased to less than 2 g/L (Fehling’s Test). Wine samples were filtered through a 0.22 μm nylon membrane for sterilization to ensure the absence of indigenous *O. oeni* strains before inoculation. MLF was subsequently performed using the native stress-tolerant strains, inoculated at a 4% (*v*/*v*) ratio in liquid medium, and incubated at 20 °C. The performance of these strains was compared to that of the superior fermentation strain SD-2a. During the process, samples were collected every 24 h to monitor the L-malic acid concentration and track the progress of MLF. The concentration of L-malic acid was determined using a highly specific L-malic acid kit (Biosystems, Barcelona, Spain) following the manufacturer’s instructions. Measurements were carried out using the Enology Y15 automatic analyzer (Biosystems, Barcelona, Spain). The color intensity was quantitatively assessed using the W100 wine color analyzer (China Hanon Co., Ltd., Jinan, China). Additionally, the physical and chemical quality parameters, including pH, ethanol content, residual sugar, total acidity, and sulfite concentration, were analyzed based on Chinese national standard test methods [54].

### 2.5. Aroma Component Analysis

The SPME parameters were performed in detail as follows: about 2 mL of the wine sample was diluted 4 times, followed by adding 2.0 g of NaCl and 20 μL of 2-octanol solution (0.016 g/L). After shocking for 15 min at 40 °C, the extraction probe was immediately inserted and extracted for 30 min. GCMS-QP2020 (Shimadzu Corporation, Kyoto, Japan) instrument and DB-WAX (60 m × 0.25 mm × 0.25) chromatographic columns were used to accomplish GC–MS analysis. The program for the heating the column’s temperature used the following conditions: the procedure was set to maintain 1 min at 40 °C, raised to 160 °C at the rate of 4 °C/min, and then increased to 220 °C at a speed of 7 °C/min with a final maintaining for 8 min. Moreover, the MS condition based on the full scan was that the ion source and the interface temperature were set to 200 °C and 220 °C, respectively [55,56]. Qualitative analysis was performed by comparing the standard aroma ingredients’ Retention Time (RT) using the NIST17 mass spectrometry library query. Quantitative analysis was carried out using 2-octanol as an internal standard and the internal standard curve method. SPSS 22 was used to analyze the data.

### 2.6. Statistical Analysis

All data were analyzed using SPSS (version 22.0; IBM, Armonk, NY, USA) for a one-way analysis of variance (ANOVA) and Duncan’s test (*p* < 0.05). Images were drawn using Origin 2022 (OriginLab Corporation, Northampton, MA, USA) and ChiPlot (https://www.chiplot.online/, accessed on 28 November 2024).

## 3. Results and Discussion

### 3.1. Strain Screening Based on PCR of Functional Genes

In this study, all 170 local isolates and 2 commercial strains of *O. oeni* were successfully analyzed for the presence of key functional genes. The β-glucosidase gene (*bgl*), esterase gene (*estA*), and glutathione reductase gene (*gshR*) were consistently amplified across all tested strains, indicating their potential functional significance. Furthermore, the presence of the *arcA*, *arcB*, and *arcC* gene clusters, which are associated with ethyl carbamate metabolism and influence wine quality and safety, was systematically investigated. Notably, the *arcA* gene was detected in all strains, signifying their capability for arginine metabolism. However, we identified 13 local isolates (6-01, 6-02, 6-05, 6-06, 6-07, 6-09, 6-10, 2-06, 1-01, 11-10, 3-01, 3-05, and 3-13) that lacked the *arcB* and *arcC* genes. Interestingly, two strains of *Leuconostoc mesenteroides*, V41 and L17, which also lacked *arcB* and *arcC*, exhibited significantly higher arginine metabolism capabilities compared to non-deleted strains. The deletion of the *arcB* and *arcC* genes disrupts the downstream catabolism of citrulline, a key intermediate in *arcA*-mediated arginine metabolism. This disruption leads to the accumulation of citrulline, which serves as a precursor for ethyl carbamate synthesis [36]. As a result, the 13 *O. oeni* strains with deletions in the *arcB* and *arcC* genes pose a potential risk for ethyl carbamate production, a critical concern for wine safety and quality. These findings highlight the importance of genetic analysis in evaluating the metabolic potential of *O. oeni* strains in winemaking environments.

A total of 172 isolated *O. oeni* strains were identified as lacking both the *odc* and *tdc* genes, indicating their inability to synthesize putrescine and tyramine [36,37,38]. Among these, 50 isolated strains (1-17, 2-15, 2-24, 3-27, 3-31, 3-35, 3-37, 3-38, 9-10, 9-13, 9-50, 9-51, 11-15, 11-55, 11-58, 11-60, 11-66, 11-69, 11-80, 11-96, 11-101, 6-07, 6-12, 5-02, 5-15, 5-16, 5-34, 5-40, 10-30, 10-31, 10-35, 10-42, 10-44, 7-04, 7-05, 7-18, 7 -23, 7-29, 8-01, 8-03, 8-06, 8-07, 8-09, 8-17, 8-26, 8-27, 8-34, 8-38, 8-39, and 8-40) with no *hdc* gene shows that these strains did not have the ability to synthesize histamine. As a result, 50 native *O. oeni* strains were successfully screened and confirmed to pose no risk for synthesizing major bioamine substances, making them suitable for food-related applications [16].

**Figure 1:**
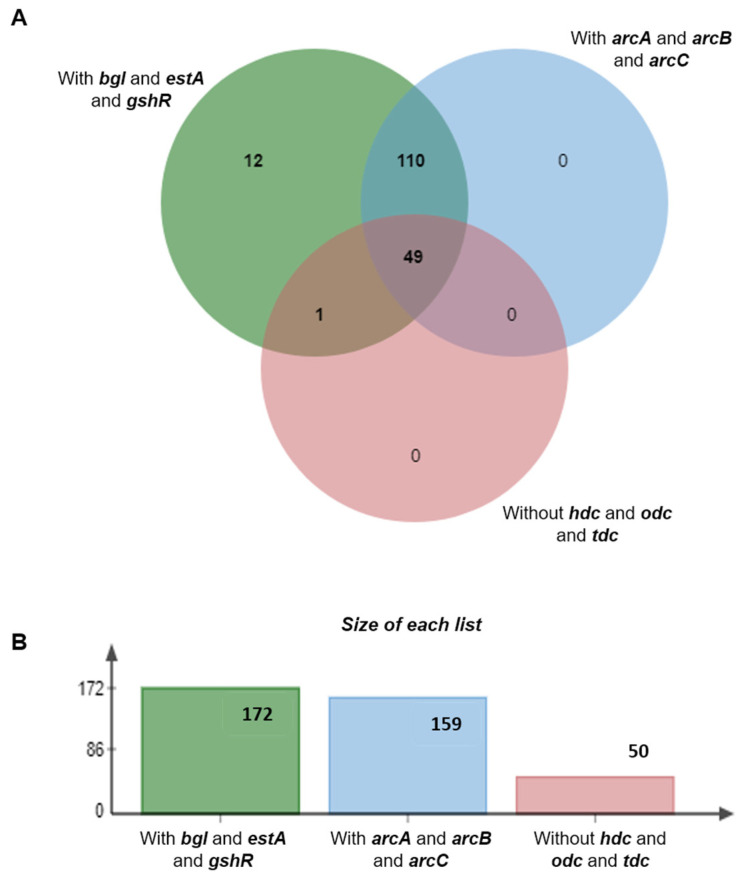
Distribution of functional and safety-related genes in the strains. (**A**) The Venn diagram of functional and safety-related genes in 172 strains; (**B**) Size of each list of functional and safety-related genes in 172 strains.

The deletion of the *arcB* and *arcC* genes in the 6-07 strain, as one of 50 isolated *O. oeni* strains, resulted in the identification of 49 indigenous strains (1-17, 2-15, 2-24, 3-27, 3-31, 3-35, 3-37, 3-38, 9-10, 9-13, 9-50, 9-51, 11-15, 11-55, 11-58, 11-60, 11-66, 11-69, 11-80, 11-96, 11-101, 6-12, 5-02, 5-15, 5-16, 5-34, 5-40, 10-30, 10-31, 10-35, 10-42, 10-44, 7-04, 7-05, 7-18, 7-23, 7-29, 8-01, 8-03, 8-06, 8-07, 8-09, 8-17, 8-26, 8-27, 8-34, 8-38, 8-39, and 8-40) that do not pose a risk of amine production (Figure 1). These strains were subsequently applied to investigate fermentation characteristics. The full identification of these strains is summarized in Table S3.

### 3.2. Screening of Superior Strains in Stress Conditions

**Figure 2:**
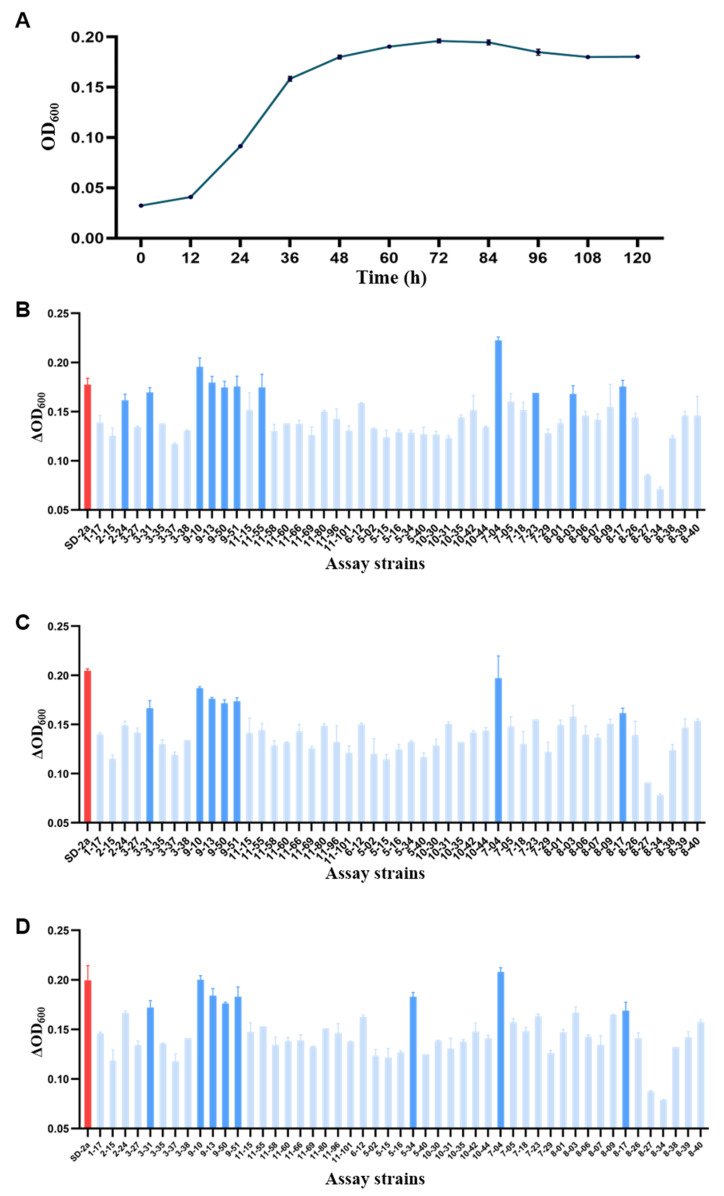
The relative growth of strains in stress-simulated wines: (**A**) SD-2a growth curve; (**B**) pH 3.2 and 14% (*v*/*v*) ethanol content; (**C**) pH 3.8 and 14% (*v*/*v*) ethanol content; (**D**) pH 3.5 and 14% (*v*/*v*) ethanol content. Dark blue indicates that all strains are not significant with SD-2a (*p* > 0.05).

High alcohol content and low pH are significant challenges during MLF [8]. In the most challenging simulated wine conditions (3.2 pH level and 14% ethanol concentration), the control strain SD-2a entered the stable phase by approximately 72 h (Figure 2A). All strains were, therefore, incubated for 72 h, and their relative growth ΔOD_600_ was assessed across various simulated wine formulations. Under the most demanding simulated wine condition (No. 9), strains 9-10 and 7-04 demonstrated superior stress resistance compared to the control strain SD-2a. Strains 2-24, 8-03, 7-23, 3-31, 11-55, 9-50, 8-17, 9-51, and 9-13 exhibited stress resistance comparable to that of the control strain SD-2a. In contrast, the remaining strains performed less effectively (Figure 2B). Therefore, A comprehensive comparative analysis was conducted on these 11 isolated strains in eight additional simulated wine stress conditions, alongside the control strain SD-2a.

In simulated wines under various stress conditions, strains 7-04 and 9-10 consistently exhibited superior stress resistance, significantly outperforming the control strain SD-2a (*p* < 0.05). Among the other nine isolated strains, their stress resistance in 14% (*v*/*v*) alcohol simulated wines No. 7 and No. 8 was generally lower than that of SD-2a. Notably, strains 9-51, 9-13, 9-50, 8-17, and 3-31 demonstrated higher stress resistance compared to strains 2-24, 8-03, 7-23, and 11-55 relative to SD-2a (Figure 2C,D). Additionally, these five strains (9-51, 9-13, 9-50, 8-17, and 3-31) showed excellent stress resistance under six other simulated wine stress conditions (*p* < 0.05) (Figure S1). These findings suggest that strains 9-10, 7-04, 9-51, 9-13, 9-50, 8-17, and 3-31 warrant further investigation for their enological characteristics in real wine environments, emphasizing their potential in enhancing malolactic fermentation processes.

### 3.3. Performance of O. oeni Strains During MLF

#### 3.3.1. Consumption of L-Malic Acid in Wines

**Figure 3:**
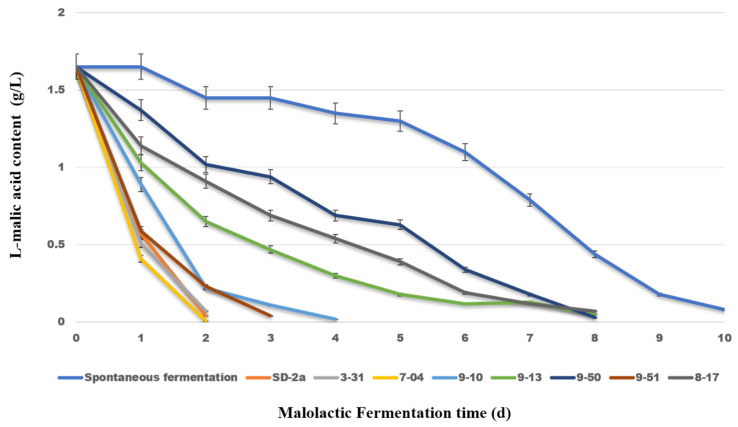
Change during MLF with different isolated strains.

These selected strains (SD-2a, 9-10, 7-04, 9-51, 9-13, 9-50, 8-17, and 3-31) were evaluated for their ability to metabolize L-malic acid during MLF. As illustrated in Figure 3, eight of the tested strains completed MLF within 2 to 8 days, all of which were quicker than the spontaneous MLF process, which required 10 days. All strains effectively reduced L-malic acid concentrations to below 0.05 g/L, with the exception of strain 8-17. Notably, strains 3-31 and 7-04, along with the control strain SD-2a, demonstrated exceptional metabolic capabilities, fully depleting L-malic acid and completing fermentation in just 2 days. Strains 9-51 and 9-10 achieved complete L-malic acid depletion in 4 days, whereas strains 9-13, 9-50, and 8-17 finished fermentation within approximately 8 days. These findings highlight the efficient degradation of L-malic acid by the indigenous strains 3-31 and 7-04, underscoring their potential for enhanced winemaking applications.

#### 3.3.2. The Change of Physical and Chemical Indexes During MLF

The physicochemical properties of Marselan wines were analyzed (Table 2), revealing notable changes post-malolactic fermentation. A significant reduction in total acidity was observed across all samples, decreasing from an initial level of 7.16 g/L to approximately 5 g/L. This substantial reduction underscores the effectiveness of MLF in modulating acidity levels, which is crucial for achieving a desirable sensory profile in the finished wine. Volatile acidity, a parameter closely tied to the wine’s sensory attributes, was found to be below the sensory threshold of 0.7 g/L, remaining well within the national standard limit of <1.2 g/L [57]. This result is particularly favorable, as it indicates that the inoculated wines maintain a pleasant flavor profile without the adverse effects of excessive volatile acidity.

While the color intensity of the wines exhibited a slight decrease, other key parameters such as alcohol content, residual sugar, and pH demonstrated minimal variation before and after MLF. This consistency highlights the selective nature of MLF, which primarily influences acidity while preserving other critical sensory and stability-related properties of the wine. These results underscore the potential of MLF to enhance the sensory profile and stability of Marselan wines, making it a valuable process in winemaking.

#### 3.3.3. Aroma Components Analysis of Superior Indigenous Strains in Marselan Wines

Over the past few decades, interest in the complexity of wine aroma has grown significantly, playing a pivotal role in consumer preferences [58,59]. MLF occurring after AF plays a crucial role in shaping the wine’s overall fermented aroma profile [60]. Wine quality and style are shaped by fermentation-derived volatiles, including alcohols, esters, and acids, which are influenced by microbial metabolism. Table 3 provides an overview of the primary volatile compounds and their respective concentrations in the inoculated Marselan wines. The aroma profiles were characterized by elevated concentrations of higher alcohols, esters, and fatty acids, which were identified and quantified using gas chromatography-mass spectrometry (GC-MS) analysis. These compounds are key determinants of the wine’s style and sensory profile, influenced in part by the microbial strains involved [17]. A significant variation in the total concentration of volatile components induced by the eight tested strains was observed, ranging from 231.72 mg/L to 303.69 mg/L. The wine fermented with strain 3-31 exhibited the highest total aroma concentration, while strain 9-13 demonstrated the lowest. Notably, the total aroma content of wines fermented with indigenous strains 3-31, 9-10, and 9-50 exceeded that of the control strain SD-2a, underscoring their potential for enhancing the aromatic complexity of Marselan wines.

The Odor Activity Value (OAV) is a critical index in sensory evaluation, calculated as the ratio of a volatile compound’s concentration to its odor perception threshold [61]. A compound with an OAV greater than 1 is considered to significantly contribute to the overall aroma [62]. In the context of Marselan wine, sensory analysis revealed that esters were the most diverse and impactful aromatic components, influenced by the microbial strains used. The total ester content varied among the samples, ranging from 47.09 mg/L to 60.02 mg/L, with the lowest concentration observed in the wine fermented with strain 7-14 and the highest in strain 3-13. Notably, strains 3-31, 9-10, 9-50, 9-51, and 8-17 produced particularly high ester concentrations. Key esters, including ethyl acetate, isobutyl acetate, ethyl butyrate, ethyl isovalerate, isoamyl acetate, ethyl hexanoate, and ethyl octanoate, had OAVs greater than 1, contributing to pleasant aromas reminiscent of apples, bananas, sweet oranges, and red berries [17,63]. While the OAV of other esters was below 1, their presence may still subtly influence the sensory profile by imparting comforting fruity and floral notes. This study underscores the role of strain selection in modulating the aromatic complexity of wines, providing insights for enhancing the sensory attributes of Marselan wine.

**Figure 4:**
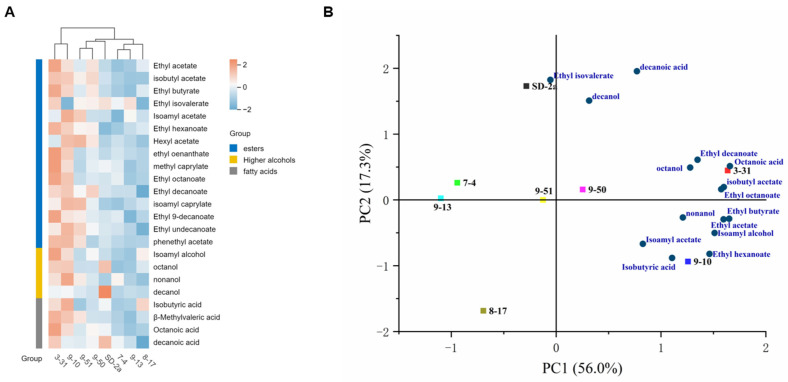
Aroma components analysis of superior indigenous strains in Marselan wines (**A**) Heatmap of aroma substances produced by different strains in Marselan wine; (**B**) PCA plot of volatile compounds in Marselan wine after fermentation with different strains.

Higher alcohols emerged as the predominant volatile compounds across all samples, accounting for over 76.2% of the total volatile profile. Their concentrations spanned a range from 177.20 mg/L to 235.44 mg/L. Notably, the sample fermented with strain 3-31 exhibited the highest concentration of higher alcohols, significantly surpassing that of the control sample. Isoamyl alcohol, the most abundant volatile compound in each sample, played a pivotal role in contributing to the wine’s mellow fragrance. Furthermore, with the exception of isoamyl alcohol, the OAV of all detected compounds remained within the range of 0.1 to 1, collectively contributing to the fruity and mellow scent profile of the wines [47,64]. Fatty acids, including isobutyric acid, octanoic acid, and decanoic acid, responsible for fruit and cheese aroma, were detected in all samples with an OAV ranging from 0.1 to 1. An appropriate fatty acid content can balance the aroma of esters in wine [65] However, elevated levels of these compounds could potentially lead to unpleasant odors, reminiscent of rot. As illustrated in the heatmap (Figure 4A), among all the wines, the sample from 9-10 exhibited the most abundant content of fatty acids, followed by the sample from 3-31. Overall, strains 3-31, 9-10, and 9-50 were identified as exhibiting excellent performance, attributed to their higher contents of esters, higher alcohols, and fatty acids in the resultant wines.

Principal Component Analysis (PCA) was employed to further explore the relationships among the volatile compounds after MLF, with the goal of distinguishing differences among the eight samples. Figure 4B illustrates the volatile compounds and their respective contributions to the PCA. The initial two principal components accounted for 73.3% of the total variance, with PC1 contributing 56.0% and PC2 17.3%. PC1 was predominantly associated with ethyl acetate, isobutyl acetate, ethyl butyrate, ethyl hexanoate, ethyl octanoate, ethyl decanoate, isoamyl alcohol, octanol, nonanol, isobutyric acid, and octanoic acid. PC2 was primarily linked to ethyl isovalerate, decanol, and decanoic acid. The positive values of PC1, representing the primary distribution of volatile compounds, effectively distinguished strains 3-31, 9-10, and 9-50. Meanwhile, PC2 facilitated the differentiation of strain SD-2a. The 9-50 strain, influenced by isoamyl acetate, octanol, and decanol, exhibited a profile of banana and rose aromas. The 3-31 strain’s flavor was characterized by a typical fruity and floral aroma, arising from a complex blend of various esters, higher alcohols, and organic acids, enriching the complexity of the wine’s scent. The predominant flavor element of SD-2a was ethyl isovalerate, which had a banana and sweet fruit fragrance, though its overall aroma was quite uniform. Strains 9-51, 7-04, and 9-13, positioned in the negative quadrant of PC1, did not positively influence the fermentation aroma of Marselan wine. These findings enhance our understanding of how indigenous strains impact wine quality and suggest potential avenues for tailored production practices.

## 4. Conclusions

In this study, we characterized 170 *O. oeni* strains, and 49 strains exhibited desirable functional properties and did not pose biosafety concerns. From these, seven strains emerged as top performers, either surpassing or matching the performance of the commercial reference strain SD-2a. Strains 3-31, 7-04, 9-51, and 9-10 completed the malolactic fermentation (MLF) process in just 4 days, highlighting their efficiency and robustness under challenging conditions. A comprehensive aroma profiling of Marselan wines subjected to MLF revealed that strains 3-31, 9-10, and 9-50 produced more intense fermentation-related volatiles. The selection of these indigenous strains not only mitigates biosafety risks but also preserves distinctive wine characteristics, thereby supporting wine biodiversity. The potential application of these strains in premium wine production and as innovative fermentation agents opens new avenues for enhancing the quality and diversity of winemaking processes. This study underscores the importance of regional microbial biodiversity in developing locally adapted winemaking solutions that contribute to the global wine industry’s innovation and sustainability. In conclusion, our findings highlight the strategic value of screening and characterizing *O. oeni* strains from distinct viticultural regions. The identified strains offer practical applications in producing high-quality wines while promoting biodiversity, thus advancing the field of enological microbiology and its contributions to sustainable winemaking practices.

## Figures and Tables

**Table 1:** The stress conditions of simulated wine.

Simulated Wine Number No.	pH	Ethanol (%(*v*/*v*))
1	3.8	10
2	3.5	10
3	3.2	10
4	3.8	12
5	3.5	12
6	3.2	12
7	3.8	14
8	3.5	14
9	3.2	14

**Table 2:** The physical and chemical index of Marselan wine before and after MLF.

Sample	Alcohol Degree %(*v*/*v*)	Residual Sugar g/L	Total Acid g/L	Volatile Acid g/L	pH	Color Intensity
Before MLF	13.43 ± 0.2 ab	3.4 ± 0.1 b	7.16 ± 0.04 a	0.30 ± 0.01 h	3.49 ± 0.04 c	9.68 ± 0.07 a
Spontaneous MLF	13.63 ± 0.14 a	2.3 ± 0.2 e	4.93 ± 0.03 e	0.57 ± 0.02 a	3.75 ± 0.1 a	7.01 ± 0.01 h
SD-2a	13.45 ± 0.03 ab	2.6 ± 0.12 d	5.24 ± 0.04 d	0.50 ± 0.02 bc	3.74 ± 0.01 a	7.16 ± 0.04 fg
3-31	13.47 ± 0.11 ab	2.0 ± 0.1 f	5.26 ± 0.02 cd	0.44 ± 0.03 de	3.64 ± 0.03 ab	7.31 ± 0.04 e
7-04	13.63 ± 0.01 a	2.0 ± 0.1 f	5.19 ± 0.04 d	0.42 ± 0.03 ef	3.68 ± 0.1 ab	7.47 ± 0.17 d
9-10	13.36 ± 0.02 b	1.6 ± 0.1 g	5.20 ± 0.1 d	0.52 ± 0.02 b	3.71 ± 0.04 ab	7.55 ± 0.05 d
9-13	13.44 ± 0.1 ab	1.6 ± 0.1 g	5.22 ± 0.06 d	0.45 ± 0.02 de	3.69 ± 0.1 ab	7.87 ± 0.05 b
9-50	13.47 ± 0.12 ab	1.9 ± 0.1 f	5.34 ± 0.04 bc	0.47 ± 0.04 cd	3.74 ± 0.04 a	7.19 ± 0.04 ef
9-51	13.40 ± 0.06 b	3.2 ± 0.1 c	5.35 ± 0.05 b	0.39 ± 0.01 fg	3.66 ± 0.02 ab	7.04 ± 0.01 gh
8-17	13.38 ± 0.08 b	3.7 ± 0.1 a	5.23 ± 0.02 d	0.39 ± 0.02 g	3.61 ± 0.03 b	7.79 ± 0.09 c

Different lowercase letters in each of these columns indicate a significant difference (*p* < 0.05), and the same letter indicates that the difference is not significant (*p* > 0.05).

**Table 3:** Concentrations, odor thresholds, and aroma descriptors of volatile aroma compounds in the wine after MLF with different strains.

RT	Compounds	Aroma Concentration (μg/L)								Thresholds	OAV	Descriptor
		SD-2a	3-31	7-04	9-10	9-13	9-50	9-51	8-17			
6.41	Ethyl acetate (mg/L)	47.78 ± 6.03 bc	57.87 ± 4.67 a	45.33 ± 0.37 c	53.13 ± 3.52 ab	46.24 ± 0.61 bc	52.96 ± 0.25 ab	49.65 ± 0.02 bc	49.87 ± 0.80 bc	7500	>1	Fruity, Sweet
9.64	Isobutyl acetate	53.98 ± 8.23 abc	61.04 ± 2.65 a	51.79 ± 1.12 bc	60.32 ± 3.35 ab	50.73 ± 3.40 c	59.28 ± 2.08 abc	57.55 ± 0.61 abc	50.85 ± 0.42 c	30	>1	Banana
10.37	Ethyl butyrate	313.7 ± 43.33 bcd	380.5 ± 23.99 a	300.82 ± 3.2 cd	355.64 ± 6.43 ab	293.13 ± 11.75 d	346.05 ± 0.13 abc	324.45 ± 1.29 bcd	324.61 ± 31.29 cd	20	>1	Strawberry
11.38	Ethyl isovalerate	7.03 ± 2.82 a	8.14 ± 3.07 a	5.29 ± 0.19 a	0.00	7.13 ± 0.63 a	6.13 ± 0.16 a	5.95 ± 0.09 a	0.00	3	>1	Strawberry, Sweet
13.32	Isoamyl acetate	684.14 ± 95.72 bc	708.86 ± 30 abc	624.39 ± 59.96 c	804.56 ± 0.8 a	726.38 ± 39.66 abc	686.85 ± 0.24 abc	774.09 ± 31.23 ab	686.12 ± 41.70 abc	30	>1	Fresh, Banana
17.59	Ethyl hexanoate	269.13 ± 56.02 d	388.54 ± 46.84 a	283.64 ± 3.64 cd	367.92 ± 14.12 ab	305.78 ± 16.36 bcd	341.45 ± 8.56 abc	338.70 ± 1.06 abc	318.01 ± 4.89 bcd	14	>1	Green apple, Strawberry
19.04	Hexyl acetate	2.15 ± 0.36 bc	2.45 ± 0.3 bc	2.35 ± 0.02 bc	3.25 ± 0.1 a	2.20 ± 0.22 bc	2.85 ± 0.69 ab	3.32 ± 0.35 a	1.97 c	1500	<0.1	Pear
21.3	Ethyl oenanthate	2.94 ± 0.43 bc	4.04 ± 0.63 a	3.07 ± 0.03 bc	3.63 ± 0.18 ab	2.94 ± 0.01 bc	3.14 ± 0.14 bc	2.93 ± 0.3 bc	2.86 ± 0.01 c	220	<0.1	Pineapple
23.38	Methyl caprylate	5.18 ± 0.62 bc	6.66 ± 0.83 a	5.00 ± 0.10 bc	5.68 ± 0.07 b	4.24 ± 0.27 c	5.06 ± 0.32 bc	4.57 ± 0.08 c	4.50 ± 0.13 c	100–400	<0.1	Sweet, orange
25.02	Ethyl octanoate	340.54 ± 38.76 c	436.27 ± 47.95 a	352.56 ± 12.69 bc	407.07 ± 23.05 ab	306.14 ± 17.15 c	345.85 ± 13.77 c	331.49 ± 7.7 c	317.24 ± 5.39 c	5	>1	Pineapple Pear, Floral
31.81	Ethyl decanoate	102.34 ± 8.11 cd	116.77 ± 5.63 ab	99.93 ± 0.39 c	119.94 ± 7.53 a	99.82 ± 2.66 c	117.68 ± 9.16 ab	108.38 ± 5.62 abc	83.36 ± 6.17 d	200	0.1~1	Fruity, Comfort
32.45	Isoamyl caprylate	6.17 ± 0.3 b	7.41 ± 0.42 ab	5.99 ± 0.06 b	8.36 ± 1.56 a	6.40 ± 0.3 b	7.03 ± 0.83 ab	8.29 ± 0.86 a	6.73 ± 0.50 ab	125	<0.1	Sweet, Cheese
33.47	Ethyl 9-decanoate	5.35 ± 0.4 bc	7.05 ± 1.25 a	5.46 ± 0.34 bc	6.07 ± 0.27 ab	4.38 ± 0.03 c	5.48 ± 0.46 bc	5.95 ± 0.56 ab	5.00 ± 0.26 bc	100	<0.1	Fruity
34.88	Ethyl undecanoate	4.21 ± 0.78 abc	4.78 ± 0.45 ab	4.12 ± 0.63 abc	5.57 ± 1.32 a	3.80 ± 0.54 bc	4.51 abc	5.13 ± 0.24 ab	3.08 ± 0.04 c	n.f	n.f	Coconut
37.08	Phenethyl acetate	12.03 ± 1.49 b	14.35 ± 0.86 a	11.55 ± 0.2 b	14.39 ± 1.11 a	11.29 ± 0.67 b	11.36 ± 0.29 b	13.35 ± 0.08 ab	11.35 ± 1.45 b	250	<0.1	Rose, Sweet
16.62	Isoamyl alcohol (mg/L)	189.40 ± 22.16 cd	235.101 ± 7.64 a	180.77 ± 0.12 d	213.13 ± 1.54 b	176.93 ± 1.22 d	202.8 ± 0.43 bc	187.78 ± 2.68 cd	206.30 ± 3.12 bc	30,000	>1	Mellow
29.02	Octanol	188.89 ± 18.6 a	185.06 ± 7.25 a	140.23 ± 1.67 b	186.12 ± 16.08 a	142.76 ± 0.66 b	155.03 ± 0.17 b	154.76 ± 3.79 b	159.75 ± 6.33 b	900	0.1~1	Rose, Organic, sweet
32.31	Nonanol	59.86 ± 7.36 cd	73.07 ± 4.27 ab	70.28 ± 1.49 bc	82.31 ± 10.02 a	60.57 ± 0.41 cd	65.42 ± 0.7 bc	71.44 ± 0.75 ab	57.21 ± 1.91 d	600	0.1~1	Orange, Strawberry
35.32	Decanol	139.40 ± 13.81 a	78.85 ± 8.12 bc	57.11 ± 1.37 d	80.82 ± 9.98 b	63.10 ± 0.24 cd	66.19 ± 0.23 bcd	71.74 ± 0.86 bcd	59.97 ± 0.8 d	400	0.1~1	Sweet floral
29.38	Isobutyric acid	3145.64 ± 174.68 ab	3390.17 ± 186.02 ab	2711.15 ± 27.26 b	3737.4 ± 811.43 a	2751.98 ± 120.88 b	2917.50 ± 38.89 ab	2668.17 ± 142.97 b	3444.28 ± 441.84 ab	8100	0.1~1	Chemical
32.67	β-Methylvaleric acid	2359.02 ± 164.93 bc	2833.93 ± 113.59 a	2256.06 ± 28.49 c	2746.89 ± 195.95 a	2150.49 ± 36.93 c	2368.88 ± 31.91 bc	2598.79 ± 142.07 ab	2346.25 ± 102.96 bc	n.f	n.f	Mint
41.88	Octanoic acid	1604.25 ± 203.46 bc	1848.35 ± 118.11 a	1490.10 ± 46.35 bc	1693.97 ± 92.80 ab	1438.23 ± 34.10 c	1627.50 ± 8.44 abc	1558.51 ± 1.71 bc	1471.46 ± 28.9 bc	8100	0.1~1	Cheese
46.28	Decanoic acid	156.69 ± 28.47 a	151.29 ± 12.5 a	132.77 ± 6.32 a	130.14 ± 11.46 ab	122.58 ± 17.54 ab	133.62 ± 9.99 a	128.96 ± 12.24 ab	96.09 ± 0.85 b	1000	0.1~1	Fatty
	Total (mg/L)	246.65	303.69	234.71	277.09	231.72	265.04	246.66	265.62			

n.f., not found. Different lowercase letters in each of these lines indicate a significant difference (*p* < 0.05), and the same letter indicates that the difference is not significant (*p* > 0.05).

## Data Availability

The data presented in this study are available on request from the corresponding author.

## Supplementary Materials

The following supporting information can be downloaded at https://www.mdpi.com/article/10.3390/foods14071207/s1. Figure S1. The relative growth of strains in stress simulated wines: (A) pH 3.8 and 10% (*v*/*v*) ethanol content; (B) pH 3.5 and 10% (*v*/*v*) ethanol content; (C) pH 3.2 and 10% (*v*/*v*) ethanol content; (D) pH 3.8 and 12% (*v*/*v*) ethanol content; (E) pH 3.5 and 12% (*v*/*v*) ethanol content; (F) pH 3.2 and 12% (*v*/*v*) ethanol content. Table S1. Genes and primers for validation. Table S2. Composition of simulated wine. Table S3. Distribution of functional and safety-related genes in the strains.
