# Supplementary material for: Bioprospecting Indigenous Oenococcus oeni Strains from Chinese Wine Regions: Multivariate Screening for Stress Tolerance and Aromatic Competence
Source: Foods. 2025 Mar 29;14(7):1207. doi: 10.3390/foods14071207 (PMC11989063; doi:10.3390/foods14071207)
Supplement: Supplementary file 1 [file foods-14-01207-s001.zip › foods-3551001-supplementary.pdf]

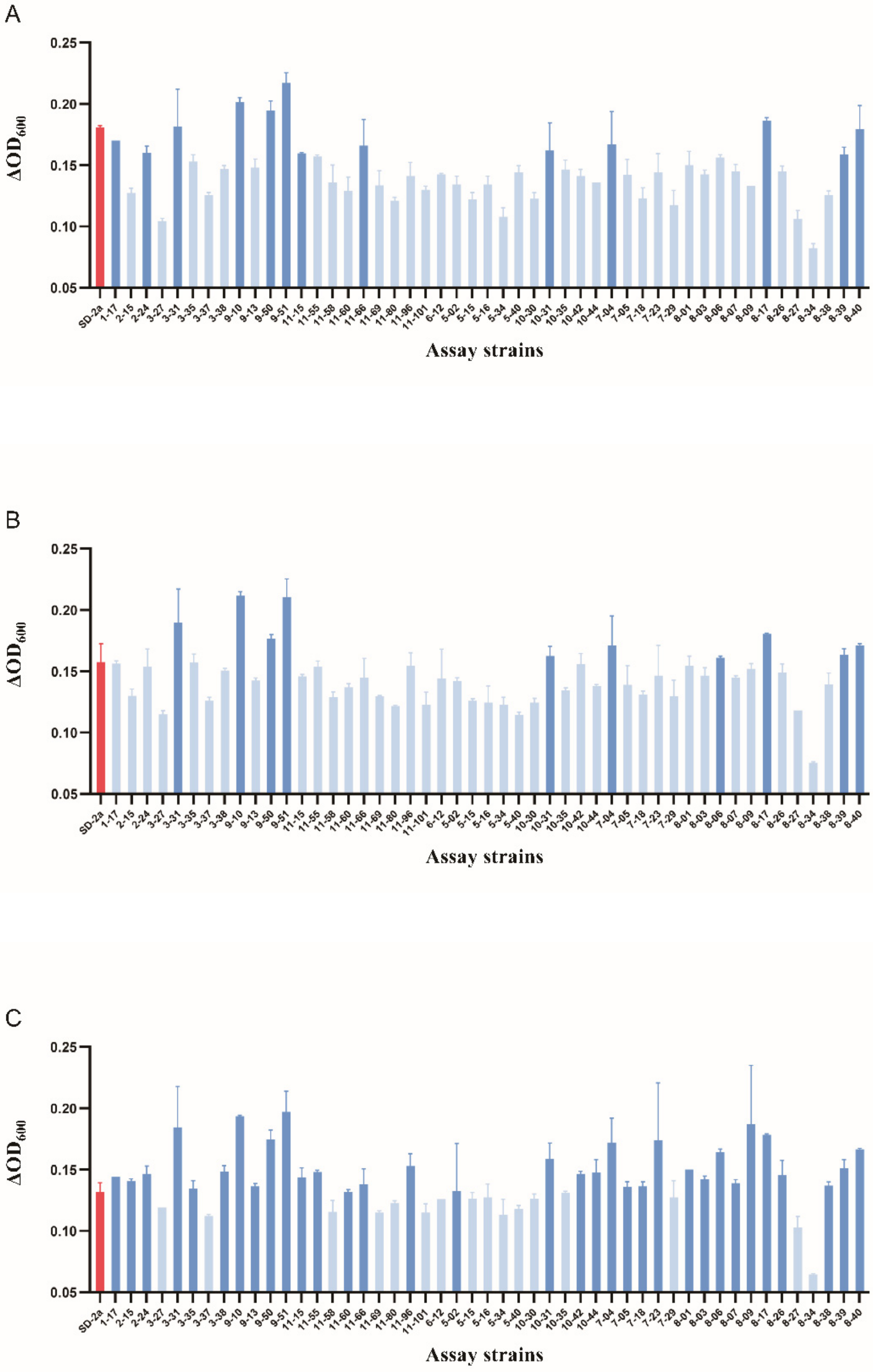
A
B
C
ΔOD600
0.25
0.20
0.15
0.10
0.05
Assay strains

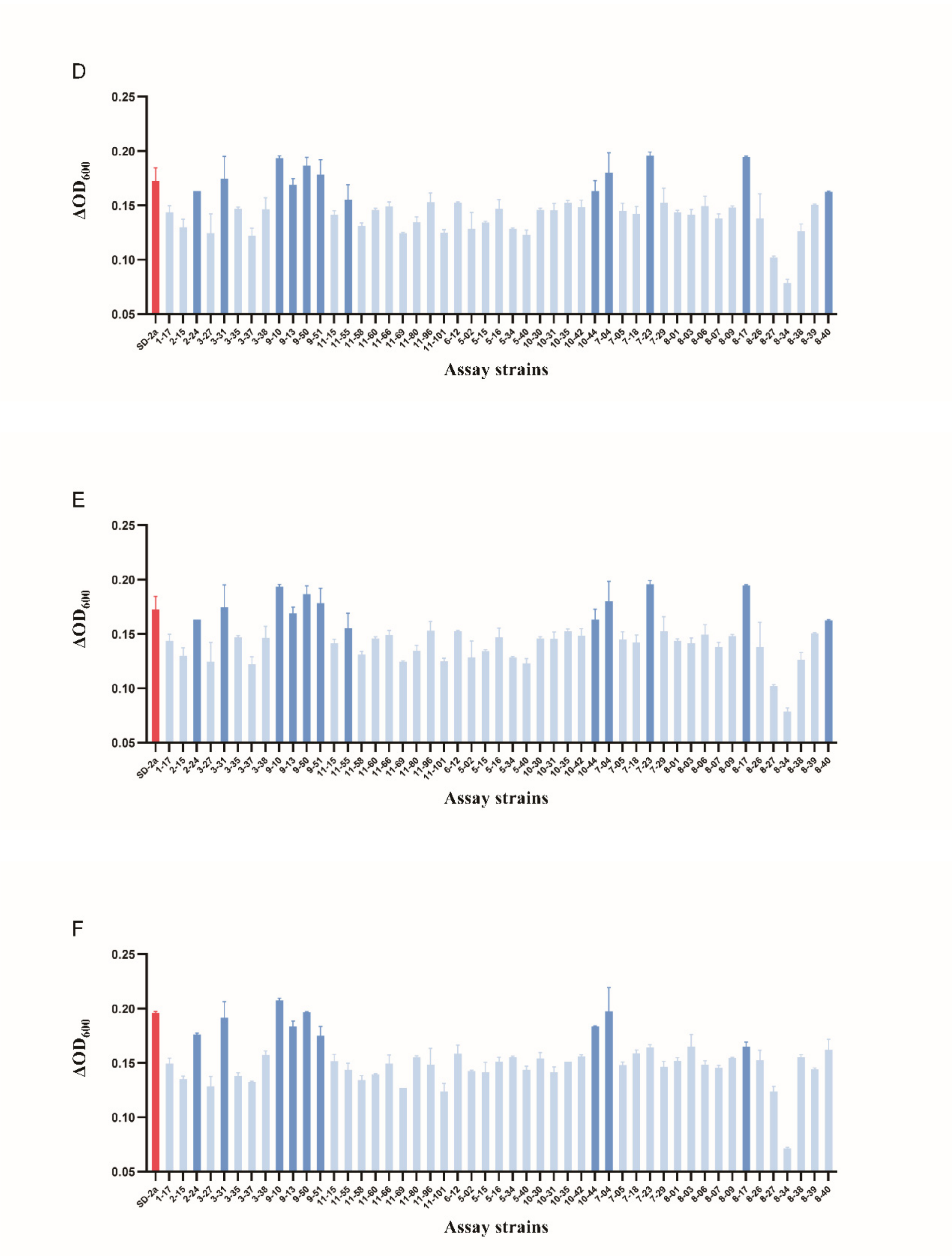

Figure S1. The relative growth of strains in stress simulated wines: (A) pH 3.8 and 10% (v/v) ethanol content; (B) pH 3.5 and 10% (v/v) ethanol content; (C) pH 3.2 and 10% (v/v) ethanol content; (D) pH 3.8 and 12% (v/v) ethanol content; (E) pH 3.5 and 12% (v/v) ethanol content; (F) pH 3.2 and 12% (v/v) ethanol content.

Table S1 Genes and primers for validation

Gene	Function	Sequence (5′ - 3′)	Size (bp)
bgl	β-Glucosidase related glycosidase	F-GATGGACCGTCTGGACTGAGAAAA<br>R-GTTAACGGCTTCGATAATCTCACTA	620
estA	Predicted esterase	F-TCCAAATTGGACAAATGACAGTTTG<br>R-ACCAGGATAATTTAAACTGGATATC	338
gshR	Glutathione reductase	F-GATATGATCGCCGGTTTGGCAAA<br>R-ATCGACGGGAAGAGATAAATCAAG	978
arcA	Arginine deiminase	F-CAYGCNATGATGCAYYTNGAYACNGT<br>R-GTRTTNSWNCCRTCRTTCCAYTGYTC	266
arcB	Ornithine Transcarbamylase	F-ATGCAYTGYYTNCCNGCNTTYCAYGA<br>R-CCNARNGTNGCNGCCATDATNGCYTT	181
arcC	Carbamate kinase	F-CAYGGNAAYGGNCCNCARGTNGGNAA<br>R-CKNCKNYANCCNCKNCCNGCRTCYTC	343
hdc	histidine decarboxylase	F-GATGGTATTGTTTCKTATGA<br>R-CCAAACACCAGCATCTTC	435
odc	ornithine decarboxylase	F-GTNTTYAAYGCNGAYAARACNTAYTTYGT<br>R-TACRCARAATACTCCNGGNGGRTANGG	1446
tdc	tyrosine decarboxylase	F-CCRTARTCNGGNATAGCRAARTCNGTRTG<br>R-GAYATNATNGGNATNGGNYTNGAYCARG	924

Table S2 Composition of simulated wine

Composition	Content
Grape	10.00 g
L-Malic acid	2.00 g
D-fructose	2.00 g
D-glucose	2.00 g
Yeast extract	4.00 g
$CaCl_2$	0.13 g
KCl	0.45 g
$(NH_4)_2SO_4$	1.00 g
$(CH_3COO)Na$	2.00 g
KH_2PO_4	0.60 g
$MgSO_4·7H_2O$	0.20 g
$MnSO_4·H_2O$	0.05 g

Table S3 Distribution of functional and safety-related genes in the strains

No.	Strain	Origin	Gene								
			bgl	*estA*	*gshR*	*arcA*	*arcB*	*arcC*	*hdc*	*odc*	*tdc*
1	SD-2a	Shandong	+	+	+	+	+	+	+	-	-
2	31-DH	IFFI	+	+	+	+	+	+	+	-	-
3	4-01	Yinchuan	+	+	+	+	+	+	+	-	-
4	4-04	Yinchuan	+	+	+	+	+	+	+	-	-
5	4-07	Yinchuan	+	+	+	+	+	+	+	-	-
6	4-10	Yinchuan	+	+	+	+	+	+	+	-	-
7	4-12	Yinchuan	+	+	+	+	+	+	+	-	-
8	4-17	Yinchuan	+	+	+	+	+	+	+	-	-
9	4-18	Yinchuan	+	+	+	+	+	+	+	-	-
10	4-23	Yinchuan	+	+	+	+	+	+	+	-	-
11	4-34	Yinchuan	+	+	+	+	+	+	+	-	-
12	4-36	Yinchuan	+	+	+	+	+	+	+	-	-
13	6-01	Yinchuan	+	+	+	+	-	-	+	-	-
14	6-02	Yinchuan	+	+	+	+	-	-	+	-	-
15	6-03	Yinchuan	+	+	+	+	+	+	+	-	-
16	6-04	Yinchuan	+	+	+	+	+	+	+	-	-
17	6-05	Yinchuan	+	+	+	+	-	-	+	-	-
18	6-06	Yinchuan	+	+	+	+	-	-	+	-	-
19	6-07	Yinchuan	+	+	+	+	-	-	-	-	-
20	6-09	Yinchuan	+	+	+	+	-	-	+	-	-
21	6-10	Yinchuan	+	+	+	+	-	-	+	-	-
22	6-11	Yinchuan	+	+	+	+	+	+	+	-	-
23	6-12	Yinchuan	+	+	+	+	+	+	-	-	-
24	6-13	Yinchuan	+	+	+	+	+	+	+	-	-
25	6-14	Yinchuan	+	+	+	+	+	+	+	-	-
26	6-15	Yinchuan	+	+	+	+	+	+	+	-	-
27	6-16	Yinchuan	+	+	+	+	+	+	+	-	-
28	5-01	Yinchuan	+	+	+	+	+	+	+	-	-
29	5-02	Yinchuan	+	+	+	+	+	+	-	-	-
30	5-11	Yinchuan	+	+	+	+	+	+	+	-	-
31	5-13	Yinchuan	+	+	+	+	+	+	+	-	-
32	5-15	Yinchuan	+	+	+	+	+	+	-	-	-
33	5-16	Yinchuan	+	+	+	+	+	+	-	-	-

34	5-17	Yinchuan	+	+	+	+	+	+	+	-	-
35	5-18	Yinchuan	+	+	+	+	+	+	+	-	-
36	5-21	Yinchuan	+	+	+	+	+	+	+	-	-
37	5-25	Yinchuan	+	+	+	+	+	+	+	-	-
38	5-28	Yinchuan	+	+	+	+	+	+	+	-	-
39	5-29	Yinchuan	+	+	+	+	+	+	+	-	-
40	5-30	Yinchuan	+	+	+	+	+	+	+	-	-
41	5-34	Yinchuan	+	+	+	+	+	+	-	-	-
42	5-38	Yinchuan	+	+	+	+	+	+	+	-	-
43	5-39	Yinchuan	+	+	+	+	+	+	+	-	-
44	5-40	Yinchuan	+	+	+	+	+	+	-	-	-
45	8-01	Yinchuan	+	+	+	+	+	+	-	-	-
46	8-03	Yinchuan	+	+	+	+	+	+	-	-	-
47	8-06	Yinchuan	+	+	+	+	+	+	-	-	-
48	8-07	Yinchuan	+	+	+	+	+	+	-	-	-
49	8-09	Yinchuan	+	+	+	+	+	+	-	-	-
50	8-17	Yinchuan	+	+	+	+	+	+	-	-	-
51	8-26	Yinchuan	+	+	+	+	+	+	-	-	-
52	8-27	Yinchuan	+	+	+	+	+	+	-	-	-
53	8-34	Yinchuan	+	+	+	+	+	+	-	-	-
54	8-38	Yinchuan	+	+	+	+	+	+	-	-	-
55	8-39	Yinchuan	+	+	+	+	+	+	-	-	-
56	8-40	Yinchuan	+	+	+	+	+	+	-	-	-
57	8-43	Yinchuan	+	+	+	+	+	+	+	-	-
58	7-01	Yinchuan	+	+	+	+	+	+	+	-	-
59	7-02	Yinchuan	+	+	+	+	+	+	+	-	-
60	7-04	Yinchuan	+	+	+	+	+	+	-	-	-
61	7-05	Yinchuan	+	+	+	+	+	+	-	-	-
62	7-18	Yinchuan	+	+	+	+	+	+	-	-	-
63	7-23	Yinchuan	+	+	+	+	+	+	-	-	-
64	7-29	Yinchuan	+	+	+	+	+	+	-	-	-
65	10-02	Yinchuan	+	+	+	+	+	+	+	-	-
66	10-06	Yinchuan	+	+	+	+	+	+	+	-	-
67	10-08	Yinchuan	+	+	+	+	+	+	+	-	-
68	10-15	Yinchuan	+	+	+	+	+	+	+	-	-
69	10-19	Yinchuan	+	+	+	+	+	+	+	-	-
70	10-21	Yinchuan	+	+	+	+	+	+	+	-	-
71	10-26	Yinchuan	+	+	+	+	+	+	+	-	-

72	10-29	Yinchuan	+	+	+	+	+	+	+	-	-
73	10-30	Yinchuan	+	+	+	+	+	+	-	-	-
74	10-31	Yinchuan	+	+	+	+	+	+	-	-	-
75	10-32	Yinchuan	+	+	+	+	+	+	+	-	-
76	10-34	Yinchuan	+	+	+	+	+	+	+	-	-
77	10-35	Yinchuan	+	+	+	+	+	+	-	-	-
78	10-36	Yinchuan	+	+	+	+	+	+	+	-	-
79	10-37	Yinchuan	+	+	+	+	+	+	+	-	-
80	10-40	Yinchuan	+	+	+	+	+	+	+	-	-
81	10-42	Yinchuan	+	+	+	+	+	+	-	-	-
82	10-43	Yinchuan	+	+	+	+	+	+	+	-	-
83	10-44	Yinchuan	+	+	+	+	+	+	-	-	-
84	10-45	Yinchuan	+	+	+	+	+	+	+	-	-
85	10-47	Yinchuan	+	+	+	+	+	+	+	-	-
86	10-48	Yinchuan	+	+	+	+	+	+	+	-	-
87	10-49	Yinchuan	+	+	+	+	+	+	+	-	-
88	10-56	Yinchuan	+	+	+	+	+	+	+	-	-
89	10-61	Yinchuan	+	+	+	+	+	+	+	-	-
90	10-69	Yinchuan	+	+	+	+	+	+	+	-	-
91	2-01	Changli	+	+	+	+	+	+	+	-	-
92	2-04	Changli	+	+	+	+	+	+	+	-	-
93	2-06	Changli	+	+	+	+	-	-	+	-	-
94	2-07	Changli	+	+	+	+	+	+	+	-	-
95	2-08	Changli	+	+	+	+	+	+	+	-	-
96	2-09	Changli	+	+	+	+	+	+	+	-	-
97	2-15	Changli	+	+	+	+	+	+	-	-	-
98	2-19	Changli	+	+	+	+	+	+	+	-	-
99	2-23	Changli	+	+	+	+	+	+	+	-	-
100	2-24	Changli	+	+	+	+	+	+	-	-	-
101	2-25	Changli	+	+	+	+	+	+	+	-	-
102	1-01	Changli	+	+	+	+	-	-	+	-	-
103	1-02	Changli	+	+	+	+	+	+	+	-	-
104	1-04	Changli	+	+	+	+	+	+	+	-	-
105	1-05	Changli	+	+	+	+	+	+	+	-	-
106	1-06	Changli	+	+	+	+	+	+	+	-	-
107	1-09	Changli	+	+	+	+	+	+	+	-	-
108	1-12	Changli	+	+	+	+	+	+	+	-	-
109	1-13	Changli	+	+	+	+	+	+	+	-	-

110	1-17	Changli	+	+	+	+	+	+	-	-	-
111	1-18	Changli	+	+	+	+	+	+	+	-	-
112	1-19	Changli	+	+	+	+	+	+	+	-	-
113	1-20	Changli	+	+	+	+	+	+	+	-	-
114	9-01	Changli	+	+	+	+	+	+	+	-	-
115	9-02	Changli	+	+	+	+	+	+	+	-	-
116	9-05	Changli	+	+	+	+	+	+	+	-	-
117	9-08	Changli	+	+	+	+	+	+	+	-	-
118	9-10	Changli	+	+	+	+	+	+	-	-	-
119	9-11	Changli	+	+	+	+	+	+	+	-	-
120	9-13	Changli	+	+	+	+	+	+	-	-	-
121	9-17	Changli	+	+	+	+	+	+	+	-	-
122	9-25	Changli	+	+	+	+	+	+	+	-	-
123	9-35	Changli	+	+	+	+	+	+	+	-	-
124	9-36	Changli	+	+	+	+	+	+	+	-	-
125	9-37	Changli	+	+	+	+	+	+	+	-	-
126	9-38	Changli	+	+	+	+	+	+	+	-	-
127	9-40	Changli	+	+	+	+	+	+	+	-	-
128	9-44	Changli	+	+	+	+	+	+	+	-	-
129	9-50	Changli	+	+	+	+	+	+	-	-	-
130	9-51	Changli	+	+	+	+	+	+	-	-	-
131	9-59	Changli	+	+	+	+	+	+	+	-	-
132	11-01	Changli	+	+	+	+	+	+	+	-	-
133	11-06	Changli	+	+	+	+	+	+	+	-	-
134	11-10	Changli	+	+	+	+	-	-	+	-	-
135	11-12	Changli	+	+	+	+	+	+	+	-	-
136	11-13	Changli	+	+	+	+	+	+	+	-	-
137	11-15	Changli	+	+	+	+	+	+	-	-	-
138	11-16	Changli	+	+	+	+	+	+	+	-	-
139	11-19	Changli	+	+	+	+	+	+	+	-	-
140	11-27	Changli	+	+	+	+	+	+	+	-	-
141	11-29	Changli	+	+	+	+	+	+	+	-	-
142	11-30	Changli	+	+	+	+	+	+	+	-	-
143	11-43	Changli	+	+	+	+	+	+	+	-	-
144	11-47	Changli	+	+	+	+	+	+	+	-	-
145	11-54	Changli	+	+	+	+	+	+	+	-	-
146	11-55	Changli	+	+	+	+	+	+	-	-	-
147	11-58	Changli	+	+	+	+	+	+	-	-	-

148	11-60	Changli	+	+	+	+	+	+	-	-	-
149	11-66	Changli	+	+	+	+	+	+	-	-	-
150	11-69	Changli	+	+	+	+	+	+	-	-	-
151	11-80	Changli	+	+	+	+	+	+	-	-	-
152	11-96	Changli	+	+	+	+	+	+	-	-	-
153	11-101	Changli	+	+	+	+	+	+	-	-	-
154	11-107	Changli	+	+	+	+	+	+	+	-	-
155	3-01	Changli	+	+	+	+	-	-	+	-	-
156	3-05	Changli	+	+	+	+	-	-	+	-	-
157	3-07	Changli	+	+	+	+	+	+	+	-	-
158	3-13	Changli	+	+	+	+	-	-	+	-	-
159	3-19	Changli	+	+	+	+	+	+	+	-	-
160	3-20	Changli	+	+	+	+	+	+	+	-	-
161	3-21	Changli	+	+	+	+	+	+	+	-	-
162	3-22	Changli	+	+	+	+	+	+	+	-	-
163	3-24	Changli	+	+	+	+	+	+	+	-	-
164	3-26	Changli	+	+	+	+	+	+	+	-	-
165	3-27	Changli	+	+	+	+	+	+	-	-	-
166	3-28	Changli	+	+	+	+	+	+	+	-	-
167	3-31	Changli	+	+	+	+	+	+	-	-	-
168	3-33	Changli	+	+	+	+	+	+	+	-	-
169	3-35	Changli	+	+	+	+	+	+	-	-	-
170	3-37	Changli	+	+	+	+	+	+	-	-	-
171	3-38	Changli	+	+	+	+	+	+	-	-	-
172	3-45	Changli	+	+	+	+	+	+	+	-	-

+ indicates successful amplification of the gene; - indicates unsuccessful amplification of the gene.
